# Label-Free and Quantitative Dry Mass Monitoring for Single Cells during In Situ Culture

**DOI:** 10.3390/cells10071635

**Published:** 2021-06-29

**Authors:** Ya Su, Rongxin Fu, Wenli Du, Han Yang, Li Ma, Xianbo Luo, Ruliang Wang, Xue Lin, Xiangyu Jin, Xiaohui Shan, Wenqi Lv, Guoliang Huang

**Affiliations:** Department of Biomedical Engineering, School of Medicine, Tsinghua University, Beijing 100084, China; suya16@mails.tsinghua.edu.cn (Y.S.); thu_frx@mail.tsinghua.edu.cn (R.F.); dwl19@mails.tsinghua.edu.cn (W.D.); yh18@mails.tsinghua.edu.cn (H.Y.); mali20020130210@163.com (L.M.); xbluo@126.com (X.L.); wangrl13@mails.tsinghua.edu.cn (R.W.); lin-x15@mails.tsinghua.edu.cn (X.L.); jin-xy17@mails.tsinghua.edu.cn (X.J.); sxh19@mails.tsinghua.edu.cn (X.S.); lvwq20@mails.tsinghua.edu.cn (W.L.)

**Keywords:** dry mass measurement, long-term cell monitoring, label-free imaging, quantitive interferometry

## Abstract

Quantitative measurement of single cells can provide in-depth information about cell morphology and metabolism. However, current live-cell imaging techniques have a lack of quantitative detection ability. Herein, we proposed a label-free and quantitative multichannel wide-field interferometric imaging (MWII) technique with femtogram dry mass sensitivity to monitor single-cell metabolism long-term in situ culture. We demonstrated that MWII could reveal the intrinsic status of cells despite fluctuating culture conditions with 3.48 nm optical path difference sensitivity, 0.97 fg dry mass sensitivity and 2.4% average maximum relative change (maximum change/average) in dry mass. Utilizing the MWII system, different intrinsic cell growth characteristics of dry mass between HeLa cells and Human Cervical Epithelial Cells (HCerEpiC) were studied. The dry mass of HeLa cells consistently increased before the M phase, whereas that of HCerEpiC increased and then decreased. The maximum growth rate of HeLa cells was 11.7% higher than that of HCerEpiC. Furthermore, HeLa cells were treated with Gemcitabine to reveal the relationship between single-cell heterogeneity and chemotherapeutic efficacy. The results show that cells with higher nuclear dry mass and nuclear density standard deviations were more likely to survive the chemotherapy. In conclusion, MWII was presented as a technique for single-cell dry mass quantitative measurement, which had significant potential applications for cell growth dynamics research, cell subtype analysis, cell health characterization, medication guidance and adjuvant drug development.

## 1. Introduction

The ability to quantitatively measure single-cell dry mass with femtogram sensitivity at the ultrastructural level is of great significance in the fields of cell growth, mass transport, cancer diagnosis and tumor therapy research. For example, there is an age-old debate about how cell growth is coordinated with cell cycle progression to maintain cell size [1]. Another example is that the necessity for active transport is especially acute, as in the case of the transport of the large objects up and down the axonal [2]. These phenomena could be better understood through quantitative mass analysis. In addition, recent evidence suggests that the disorder strength of nanoscale architecture increases in microscopically normal-appearing cells outside of tumors, which implicates that cell dry mass analysis can be used to identify patients harboring malignant cells [3,4]. Moreover, it has been reported that gene transcription patterns can be controlled by modulating the scaling of chromatin-packing density within the nucleus [5]. This indicates a new alternative tool for the screening of chemotherapeutic adjuvants. All of these biomedical studies are related to single-cell dry mass quantitation.

However, quantitative cell dry mass measurement still needs further refinement. Currently, Godin et al. have quantified the buoyant mass of cells by shifts in the resonant frequency of vibrating microchannels [6]. Although the sensitivity is high, it is not suitable for adherent cells and long-term monitoring. Park et al. measured cell biophysical properties using an array of micro-electro-mechanical system resonant mass sensors [7]. The sensitivity is impacted and influenced by adherent cell elasticity and viscosity. In contrast, Popescu et al. developed a unique imaging method, spatial light interference microscopy, that continuously performs parallel cell growth measurement over more than one cell cycle, which can be applied equally well to adherent and non-adherent cells [8,9]. However, their analysis lacks ultrastructure analysis. Thus, label-free and long-term techniques for single-cell dry mass monitoring require further development.

The label-free imaging technique has attracted much attention [10,11]. Especially, quantitative phase imaging (QPI) focuses on precisely quantifying the phase shift caused by heterogeneous specimens [12,13]. Over the past decade, a number of novel QPI platforms have been developed to improve imaging resolution and sensitivity both in time and space, which enables a range of applications, including monitoring the dynamics of single cells, medical diagnosis and neuroscience studies [14,15,16]. However, current QPI technology still has the following problems: (1) Phase wrapping occurs in some QPI technologies, which leads to periodic phase interference in dry mass measurement and the small measurement period [17,18]. (2) Cellular dry mass measurement accuracy is affected by nanoscale structure fluctuations because the refractive index (RI) is coupled with nanoscale thickness. (3) Current QPI imaging platforms do not allow quantitative liquid or drug dosing without disturbance during in situ imaging. The movement of cells causes changes to system calibration parameters and the interruption of imaging. The problems mentioned above urgently need to be solved.

Here, we propose a label-free, multichannel, wide-field interferometric imaging (MWII) technique to quantitatively measure single-cell dry mass with femtogram sensitivity, which can reveal intrinsic cellular conditions at the ultrastructural level during long-term culture. Compared to comparable techniques, the proposed method possesses three major merits. First, multiple illumination wavelength modification extends the measurement period and avoids phase wrapping, which is a common problem in QPI techniques. Further, cellular dry mass measurement accuracy was not affected by nanoscale structure fluctuations because the thickness and RI information were independently reconstructed with the ergodic method. In addition, a customized compact cell incubator was developed to culture living cells for in situ measurement. With the proposed method and imaging system, different dry mass growth characteristics between HeLa cells and Human Cervical Epithelial Cells (HcerEpiC) were revealed. The relationship between therapeutic effects and cell heterogeneity was analyzed. We found that MWII has significant potential applications for cell growth dynamics research, cell-level diagnosis, cell metabolism detection and cellular health characterization.

## 2. Materials and Methods

### 2.1. Quantitative Dry Mass Imaging

**Figure 1:**
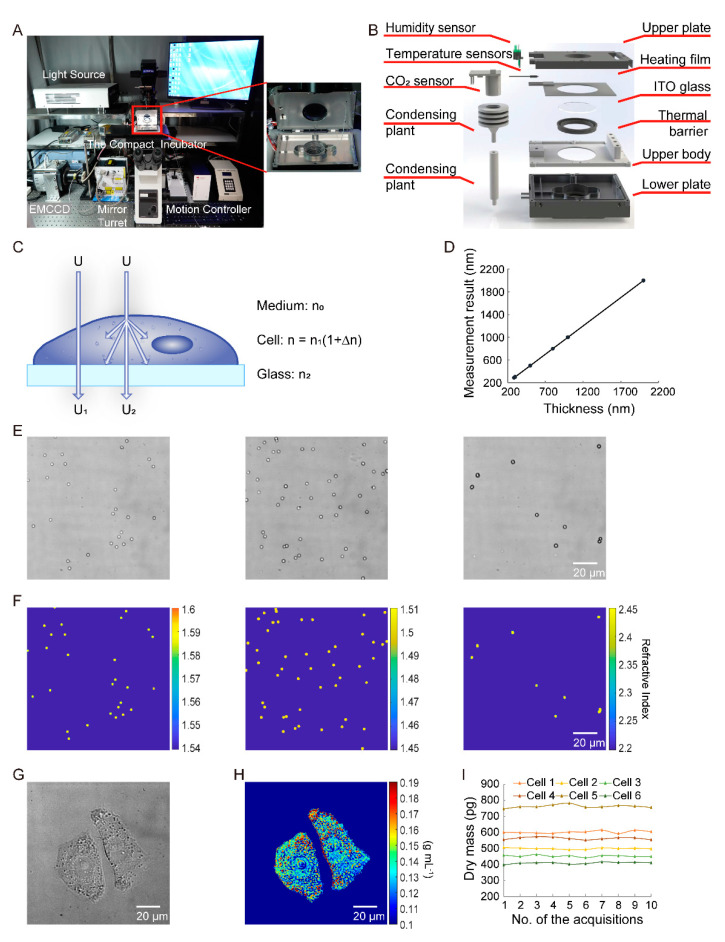
The theoretical principle of multichannel wide-field interferometric imaging (MWII) technique. (**A**) The imaging system. (**B**) The structure of the compact incubator. (**C**) The mathematical description of the light propagation. (**D**) Wafer thickness measurement results. (**E**) Imaging results of polystyrene, polymethyl methacrylate and iron oxide microspheres with differential interference contrast (DIC) microscopy. (**F**) Reconstruction results of microspheres with MWII, in which the quantitative colors indicate the refractive index of the microspheres. (**G**) Imaging results of fixed HeLa cells with DIC microscopy. (**H**) Reconstruction results of fixed HeLa cells with MWII, in which the quantitative colors indicate the density of the cells. (**I**) The dry mass measurement results of six fixed single HeLa cells after 10 acquisitions successively.

The imaging system consists of a home-assembled, wide-filed inverted microscope and a compact cell incubator, which is shown in Figure 1A. A cool white-light-emitting diode (LED) (LED 100, Marzhauser, Wetzlar, Germany) was used as an illuminating, spatially coherent light source. An X60 air-coupled objective lens (NA = 0.7) (LUCPLFLN60X, Olympus, Tokyo, Japan) was utilized for the collection parts of the setup. The resolution and the field of view of the objective lens were 0.48 μm and 1.42 × 104 μm^2^, respectively. The collimated beam transmitted a sample and then collected it by the objective lens. A filter wheel (Lambda 10-B, Sutter, Novato, CA, USA) with filters of 447, 525, 593 and 692 nm was assembled between the optical pathway of the lens and the image plane. The imaging device utilized in the system was an electron multiplying charge-coupled device (EMCCD) (Ixon Ultra, Andor, Oxford, UK) with a pixel size of 16 μm. The temperature, carbon dioxide concentration and humidity of the compact incubator, which is illustrated in Figure 1B, were controlled precisely for long-term cell culture. The culture performance comparison of the customized incubator and the commercial incubator is shown in Supplementary Figure S1 (see Section S1: The Customized Incubator in Supplementary Materials for more details about the customized incubator).

The theoretical principle of MWII is as follows (Figure 1C): living cells were immersed in the culture medium and grew on a glass substrate. $n_{0}$ and $n_{2}$ represent the mean RI of the culture medium and the glass, respectively. The living cells can be regarded as a spatially inhomogeneous sample with RI distribution $\left[n=n_{1}(1+\Delta n(z))\right]$, which is a function of location z. $n_{1}$ is the average RI of living cell cytoplasm, which has been widely reported in the latest literature. The mean and standard deviation of RI in these papers are 1.36 and 0.01. Therefore, the mean RI value of 1.36 was utilized as the average RI of cell cytoplasm in this work [19,20,21,22]. Assuming U is the illumination field, the illuminating light was normally incident onto the sample, and the absorption of the living cells was negligible. Finally, the field that reaches the image plane of an epi-illumination bright-field microscope is a result of optical interference between (i) the field transmitted through the sample without scattering (referred to as reference beam $U_{1}$) and (ii) the field scattered from its internal fluctuations (scattering beam $U_{2}$), with only the waves propagating at solid angles within the NA of the objective were collected. Thus, the intensity of the interference signal equals [23]: $I\left(\lambda \right)={\left|U_{1}\left(\lambda \right)+U_{2}\left(\lambda \right)\right|}^{2},$ where λ is the wavelength and I(λ) is the coherent intensity of the corresponding wavelength. The four wavelengths of the illuminating light were 447, 525, 593 and 692 nm. According to the Fresnel coefficient and optical phase theory [12,24], $U_{1}\left(\lambda \right)$ and $U_{2}\left(\lambda \right)$ equal:

(1) $$U_{1}\left(\lambda \right)=\frac{4n_{0}n_{1}}{\left(n_{0}+n_{1}\right)\left(n_{1}+n_{2}\right)}U\left(\lambda \right),$$

(2) $$U_{2}\left(\lambda \right)=\frac{4n_{0}n_{1}}{\left(n_{0}+n_{1}\right)\left(n_{1}+n_{2}\right)}\frac{2\pi }{\lambda }n_{1}\int^{\;}\Delta \mathrm{nsin}\left(\frac{4{\pi n}_{1}\left(z+\Delta z\right)}{\lambda }\right)d_{z}U\left(\lambda \right),$$

where z represents the distance between the focal plane and the glass, to which the living cells are attached. This axial position could be roughly recorded with an axial objective moving stage. Δz represents the modification value of the focal plane. Thus, the coherent intensity of the image plane is:

(3) $$I\left(\lambda \right)=\frac{16n_{0}{}^{2}n_{1}{}^{2}}{{\left(n_{0}+n_{1}\right)}^{2}{\left(n_{1}+n_{2}\right)}^{2}}U{\left(\lambda \right)}^{2}+\frac{32n_{0}{}^{2}n_{1}{}^{2}}{{\left(n_{0}+n_{1}\right)}^{2}{\left(n_{1}+n_{2}\right)}^{2}}\frac{2\pi }{\lambda }n_{1}\int^{\;}\Delta \mathrm{nsin}\left(\frac{4{\pi n}_{1}\left(z+\Delta z\right)}{\lambda }\right)d_{z}U{\left(\lambda \right)}^{2}.$$

As the sample is weakly scattering, $O\left(U_{2}{\left(\lambda \right)}^{2}\right)$ is neglected here [18]. The values of Δn and accurate axial position Δz could be both calculated with the ergodic method. As Equation (3), the values of Δn and Δz could be obtained simultaneously within a reasonable range. In this work, the ergodic range of Δn was set to be −0.3 to 0.3 with the ergodic step of 0.0001. Additionally, the ergodic range of Δz was −300 nm to 300 nm with the ergodic step of 0.1 nm. The error between the actual and theoretical coherent intensity of the image plane is a monotone function of Δn and Δz. The minimum error corresponds to the optimal values of Δn and Δz. After correction of the nanoscale axial fluctuation of the scattering signal with the ergodic method, the dry mass measurement accuracy is greatly improved. The numerical simulation diagrams of ergodic method and the accuracy comparison between one and multiple wavelength measurements are shown in Supplementary Figure S2 (see Section S2: The Accuracy Improvement with Multiple Wavelength Measurements in supplementary Materials for more details about the ergodic method and the accuracy improvement).

Along the optical axis detection, cells were scanned with 0.1 µm slice spacing and 5 µm total path. The cell dry mass density maps of the in-focus slices were evaluated using the Gladstone–Dale relation: $\rho =\left(n-n_{w}\right)/\alpha$ [12], where $n_{w}$ is the RI of water, and α is the specific refractive increment (0.18 mL × g^−1^). The total dry mass was then calculated by integrating the region of interest.

### 2.2. Image Segmentation

To enable automatic cell nucleus segmentation, a custom MATLAB image process code was designed. Briefly, visualizing the nucleus of living cells was implemented using a synthetic fluorescent DNA probe of Hoechst (Ab228550, Abcam, Cambridge, UK) [25]. Then, we used ROI management of ImageJ v2 to extract and compile statistics characteristics of the nucleus. The RI of the nucleus tended to be >1.42, and the value was consistent with previous literature reports [20]. Thus, this value was chosen as the segment threshold, and it is noted that Hoechst was not utilized in subsequent segmentation operations. However, due to the cells’ complicated morphology, there were always some pixels recorded at an abnormal RI in both the nucleus and cytoplasm. We used this experience threshold to obtain binary masks and then performed erosion operations to remove the small size area. In the segment masks, positive pixels with a RI > 1.42 were set to one, while negative pixels were set to zero. Next, the edges of the positive area were determined with the Canny operator edge detection method. Then, we conducted dilation operations and merely maintained the dilation results inside the edges to compensate for the pixels with less threshold in the nucleus. Last, we multiplied the dry mass reconstruction results with the binary masks to calculate the dry mass of the nucleus. Similarly, the cytoplasm dry mass distribution could be calculated with the reverse masks.

### 2.3. Preparation

In this work, silicon wafers with different thicknesses were cleaned with the piranha solution before being repeatedly thickness measurement 10 times for each thickness [26]. The proposed method sample was validated on three engineered samples: 500 nm diameter polystyrene microspheres (n = 1.59), polymethyl methacrylate microspheres (n = 1.50) and iron oxide microspheres (n = 2.42). The RI of 200 microspheres for each material was measured and counted. HeLa cells (HeLa S3, contain the *HPV-18* sequence, positive for keratin) and HCerEpiC (characterized by immunofluorescence with antibodies specific to vWF/Factor VIII and CD31 (PECAM), negative for HIV-1, HBV, HCV, mycoplasma, bacteria, yeast, and fungi) were cultured and fixed using general methods [8,9]. For living cell measurement experiments, the dry mass and size (pixels in the image) of HeLa cells were measured when cells were ~90% confluent. Then, 1 µL of a 25% NaCl solution was pumped into the Petri dish within the compact cell incubator using a micropump (HALMA, Havant, UK). Prior to that, 1 µL of culture medium was pumped out of the dish to maintain a constant culture volume of 1 mL. The cell dry mass and size were measured at this hypertonic condition. After perfusion and measurement were repeated nine times, the cells were washed twice with PBS and then perfused with 1 mL complete medium. Cell dry mass and size were measured again at this physiological osmotic pressure. The whole experiment could be performed within 15 min so that the changes in cell-intrinsic living status could be ignored.

For long-term cell growth monitoring experiments, HeLa cells and HCerEpiC were cultured in the compact incubator, and a 2.65 mm × 2.65 mm area was scanned every 2 h for 36 h. The overall health of the culture was evident by the consistent growth of the cells. The growth dynamics of the full cell cycle could be measured from a single cell as it divides into two cells and then daughters into four. For quantitative analysis of single-cell heterogeneity experiments, HeLa cells were cultured for 24 h in serum starvation medium for cell cycle synchronization. Then, the dry mass, dry mass of the nucleus, nuclear–cytoplasmic ratio and density standard deviation of the nucleus for the synchronized single cells were measured with the proposed method. Subsequently, the cells were treated with the half-maximal inhibitory concentration (IC50) of Gemcitabine as 38 μM for 24 h, during which some of the cells died, whereas others survived. Cell viability was evaluated based on the morphology and fluorescent results of cells. Cells with incomplete membrane or floating cells were considered dead. Additionally, cells with indistinguishable morphology were determined by Calcein-AM/PI double staining. Cells that fluoresced green were treated as living cells, while cells fluoresced red were treated as dead cells [27]. The relationship between the single-cell heterogeneity and chemotherapeutic efficacy could be revealed.

## 3. Results

### 3.1. Principle Verification

In this experiment, the sensitivity of the proposed method was determined. First, silicon wafers with different thicknesses were exploited to verify the measurement principle of the system. The thickness measurement results and the theoretical values are shown in Figure 1D. The theoretical values were measured by the method proposed in the previous literature [28]. For the 285 nm silica layer, the measurement was 285.00 ± 0.81 nm. For the 300 nm silica layer, the measurement was 300.49 ± 0.78 nm. For the 500 nm silica layer, the measurement was 500.00 ± 0.81 nm. For the 800 nm silica layer, the measurement was 799.99 ± 0.79 nm. For the 1000 nm silica layer, the measurement was 999.01 ± 0.81 nm. For the 2000 nm silica layer, the measurement was 1999.00 ± 0.80 nm. The coefficient of determination was 0.999. The maximal measurement error was 1.00 nm, and the maximal standard deviation was 0.80 nm. Thus, the sensitivity of thickness was 2.40 nm, which was three times the standard deviation, and the optical path difference (OPD) sensitivity of MWII reached 3.48 nm, which was the thickness sensitivity multiplied by the average RI of silica. Thus, the sensitivity of dry cell mass was 0.97 fg (See Section S3: The Sensitivity of Dry Cell Mass in Supplementary Materials for more details about the derivation process).

Next, the accuracy of the MWII was validated. The RI of polystyrene microspheres, polymethyl methacrylate microspheres and iron oxide microspheres were measured using the proposed method. The mean and standard deviation of RI for polystyrene microspheres were 1.5846 and 0.0033. The mean and standard deviation of RI for polymethyl methacrylate microspheres were 1.4935 and 0.0009. The mean and standard deviation of RI for iron oxide microspheres were 2.4132 and 0.0077. The maximal relative change (maximum change/average) in RI was 0.4% and the maximal standard deviation was 0.0077. The imaging results with differential interference contrast (DIC) microscopy and imaging reconstructed results with MWII of the microspheres are shown in Figure 1E,F, in which the quantitative colors indicate the RI of the microspheres. Moreover, the RI measurement noise would affect the accuracy of dry mass measurement in our system. The potential degeneration was demonstrated in Supplementary Materials (See Section S4: The Noise of Dry Mass Measurement in Supplementary Materials for more details). 

Then, the repeatability of MWII was verified. We measured the dry mass of 200 fixed HeLa cells after 10 successive acquisitions for each cell. Figure 1G and H shows an example of the imaging results with DIC microscopy and the imaging reconstruction results with MWII for two fixed cells, in which the quantitative colors indicate the density of the cells. It was obvious that MWII enabled the quantitative imaging of the transparent samples, whereas traditional microscopy only offered qualitative information. The dry mass standard deviations of these two cells were 5.57 and 7.32 pg, the maximum relative changes (maximum change/average) in dry mass were 2.7% and 2.0%, respectively. Figure 1I shows an example of the dry mass measurement results of six fixed single cells. The average maximum relative change in dry mass of the 200 cells was 2.4%. Besides, the effect of field position on repeatability was analyzed. Thirty of the fixed HeLa cells were utilized, and each of them was imaged at 25 different positions within the field of view. Then, the cellular dry mass results of each cell were calculated and analyzed. The maximal relative change in the dry mass of a single fixed cell due to field position was 3.5%. These results indicated that MWII has excellent stability.

### 3.2. Quantitative Dry Mass Measurement of Living Cells at the Ultrastructural Level

**Figure 2:**
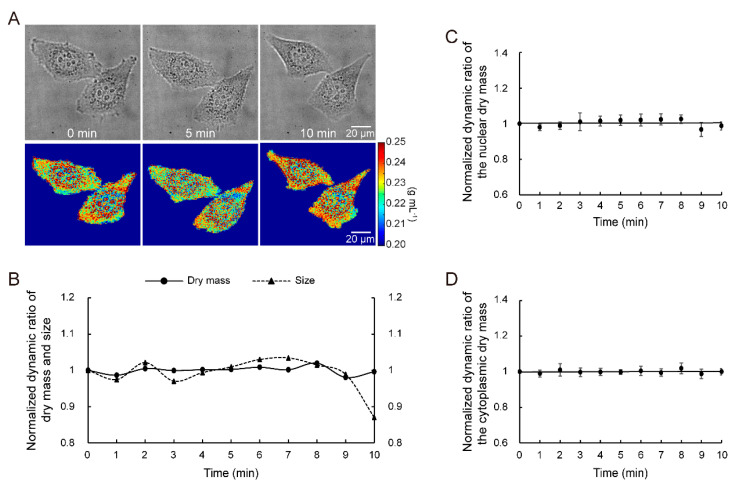
Quantitative dry mass measurement of live cells during osmotic pressure change. (**A**) Imaging reconstruction of two living HeLa cells during osmotic pressure change. (**B**) Normalized dynamic ratio of dry mass and size for 200 living single cells. Normalized dynamic ratio of the nuclear (**C**) and cytoplasmic (**D**) dry mass for the 200 cells.

In this experiment, the ability of MWII to measure the intrinsic status of living cells under varied and complicated culture conditions was demonstrated. The dry mass and size of 200 living HeLa cells were measured in an isotonic medium at 0 min and then in hyperosmotic solution during 1–9 min. At 10 min, cells were returned to isotonic conditions and measured. The change in cell-intrinsic living status could be ignored because the experiment was finished within 15 min. Figure 2A shows an example of the imaging results with DIC microscopy and imaging reconstruction results with MWII at 0, 5 and 10 min for two living HeLa cells. The quantitative colors indicate the density of the cells. In Figure 2B, the normalized dynamic ratio of dry mass and size measurement results, with respect to the value at 0 min, of the 200 single cells are shown. The average maximum relative change in cell size (12.3%) is far more than that of cell dry mass (3.7%), which proved that MWII could monitor intrinsic cell status despite environmental changes. The analysis of the single-cell dry mass at the ultrastructural level also supported this conclusion. The normalized dynamic ratios of nuclear and cytoplasmic dry mass are shown in Figure 2C,D. Both of the average maximum relative changes in dry mass were <5.0%. In addition, The normalized dynamic ratio of dry mass and size for 100 living single HeLa cells at hypoosmotic conditions is shown in Figure S3. The ability of MWII to measure the intrinsic status of living cells under hypoosmotic conditions was also validated by the less-average maximum relative change in dry cell mass (3.3%) than size (27.4%) (see Section S5: Quantitative Dry Mass Measurement of Living Cells at Hypoosmotic conditions in Supplementary Materials for more details).

### 3.3. Long-Term Dry Mass Monitoring of HeLa Cells and HCerEpiC during Cell Growth

**Figure 3:**
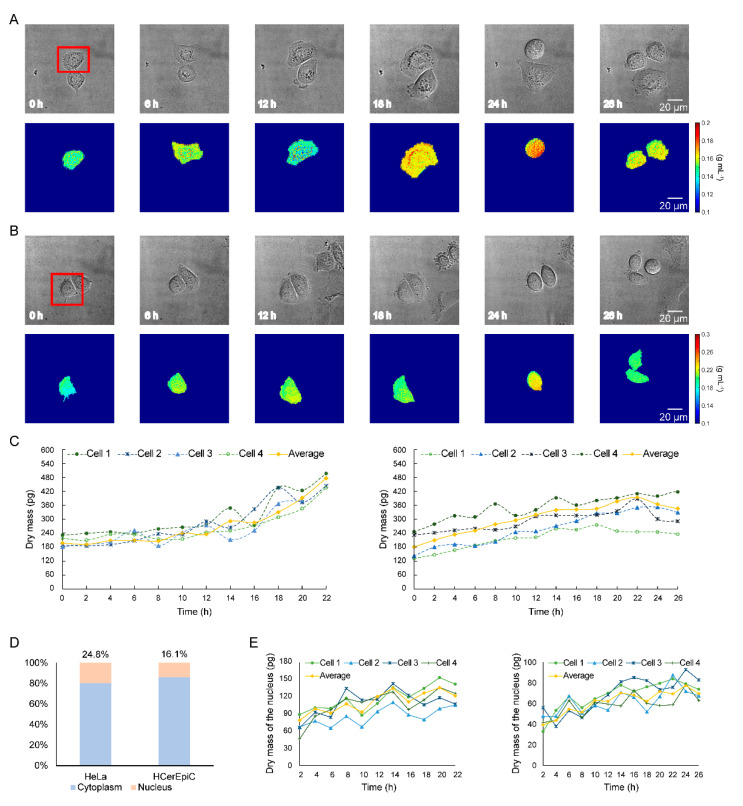
Long-term and quantitative dry mass monitoring of living HeLa cells and Human Cervical Epithelial Cells (HCerEpiC) during a cell cycle. Imaging results with DIC microscopy and reconstruction results with MWII for the single HeLa cell (**A**) and Human Cervical Epithelial Cell (**B**) indicated by a red box during a cell cycle. (**C**) Dry mass measurement and statistic results of single HeLa cells (left) and HCerEpiC (right) before M phase. The yellow curves represent the average dry mass measurement and statistic results of 98 HeLa cells and 101 HCerEpiC. (**D**) The average nuclear-cytoplasmic ratio of HeLa cells and HCerEpiC. (**E**) Dry mass of the nucleus for 4 single HeLa cells (left) and HCerEpiC (right). The yellow curves represent the average nuclear dry mass measurement and statistic results of 98 HeLa cells and 101 HCerEpiC. The values at 0 h were discarded due to the invisible nuclei.

In this experiment, the different cell growth characteristics between HeLa cells and HCerEpiC were monitored. Figure 3A,B shows the imaging results with DIC microscopy and imaging reconstruction results with MWII for the single HeLa cell and HCerEpiC, indicated by a red box during a cell cycle. The quantitative colors indicate the density of the cells, and the range of the density in each image is the same for a better visual effect. The single cell expanded and then contracted before it divided. In Figure 3C, the statistical mean growth curves of 98 HeLa cells and 101 HCerEpiC before the M phase are shown, as well as four examples of single-cell growth curves. The differences in cell growth can be seen clearly. The dry mass of HeLa cells consistently increased before the M phase, whereas that of HcerEpiC increased and then decreased. There were two typical phases in dry mass growth curves before mitosis. For HeLa cells, the average growth rate of the first phase was 5.3%, which was relatively flat. The average growth rate of the second phase was 19.7%, which indicates a rapid growth due to the synthesis of a large number of substances for cell division. However, the growth pattern of HCerEpiC was very different. In the first phase, the average cell growth rate was 8.0%. In the second phase, the dry mass of the cells decreased, and the growth rate was −6.9%.

We also analyzed measurement results at the ultrastructure level. As shown in Figure 3D, the mean nuclear–cytoplasmic ratio of HeLa cells was 8.7% greater than that of HCerEpiC. The increase of dry cell mass was mainly contributed by the increase of dry mass in the cytoplasm. The dry mass of the nucleus fluctuated during a cell cycle. Figure 3E shows the curves of dry nuclear mass for four single HeLa cells (left) and HCerEpiC (right) during a cell cycle before the M phase. Additionally, the curves of average nuclear dry mass for 98 HeLa cells and 101 HCerEpiC are shown. There were usually three typical peaks in the curves.

### 3.4. Quantitative Analysis of Single-Cell Heterogeneity in Dry Mass

**Figure 4:**
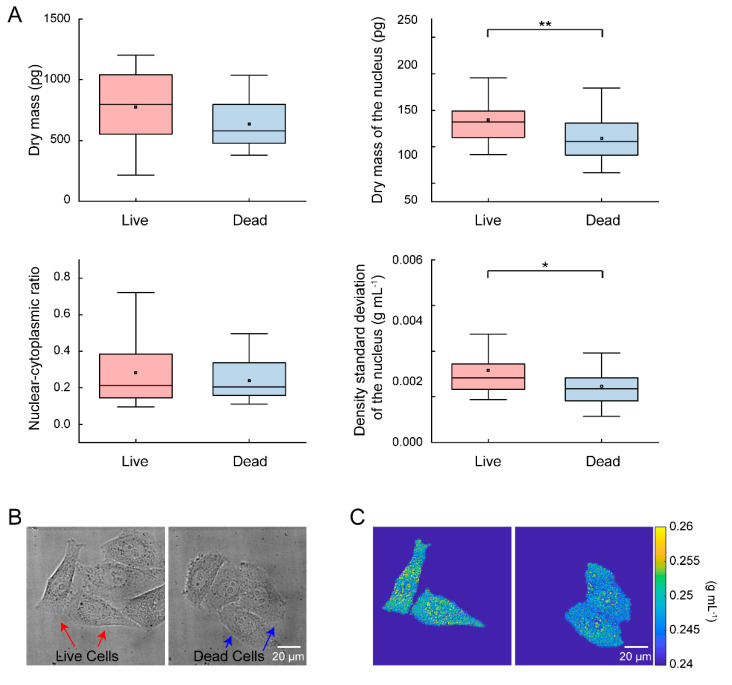
The relationship between cell heterogeneity and chemotherapeutic efficacy for single HeLa cells. (**A**) The dry mass, dry mass of the nucleus, nuclear–cytoplasmic ratio and density standard deviation of the nucleus for 150 living single cells and 149 dead single cells. ** *p* < 0.01, * *p* < 0.05. (**B**) Imaging results with DIC microscopy before dosing. The red arrow indicates the living cells, and the blue arrow indicates the dead cells. (**C**) Imaging reconstruction results before dosing with MWII.

In this experiment, the relationship between single-cell heterogeneity and chemotherapeutic efficacy was revealed. Synchronized HeLa cells were monitored using MWII before being treated with Gemcitabine. Cells that were subsequently susceptible or resistant to the treatment were assigned as dead or live cells group. The dry mass, dry mass of the nucleus, nuclear–cytoplasmic ratio and the density standard deviation of the nucleus were compared between 150 living single cells and 149 dead single cells and are shown in Figure 4A.

The mean dry mass and nuclear–cytoplasmic ratio of the living single cells were 775.58 pg and 0.28, respectively. In contrast, the mean dry mass and nuclear–cytoplasmic ratio of the dead single cells were 636.13 pg and 0.24. The results displayed that the dry mass and nuclear–cytoplasmic ratios of the single cells had no significant correlation with chemotherapeutic efficacy (*p* > 0.05). However, the mean nuclear dry mass and nuclear density standard deviation of the living single cells were 136.72 pg and 0.0032 g × mL^−1^, respectively. In contrast, the mean nuclear dry mass and nuclear density standard deviation of the dead single cells were 111.52 pg and 0.0025 g × mL^−1^, respectively. Thus, the single cells with higher nuclear dry mass (*p* < 0.01) and higher nuclear density standard deviations (*p* < 0.05) were more likely to survive the chemotherapy. Figure 4B shows the imaging results with DIC microscopy before dosing. There are four selected synchronized cells in the images. The red arrow indicates two live cells, and the blue arrow indicates two dead cells. Figure 4C shows the imaging reconstruction results before dosing with MWII, in which the quantitative colors indicate the density of the cells. For the two live cells and the two dead cells, the dry nuclear masses were 141.50, 137.97, 72.58 and 69.28 pg; the nuclear density standard deviations were 0.0033, 0.0038, 0.0017 and 0.0018 g × mL^−1^.

## 4. Discussion

In this work, MWII was proposed for femtogram cell dry mass measurements. The main merit of the multiple illumination scheme is to extend the measurement period and avoid phase wrapping. The measurement period is λ/2n (λ represents illumination wavelength, n represents RI of samples) in the case of phase wrapping, which is usually less than 1 µm [29]. However, the measurement periodicity of this method is multiple products of the four wavelengths and divided by two, which is 4.8 × 10^7^ μm. This value is far more than common cell thickness. Furthermore, based on the multichannel information, RI value and nanoscale axial fluctuation of the scattering signal could be both calculated with the ergodic method, and this advantage helps to improve the measurement accuracy. The accuracy comparison between one and multiple wavelength measurements is illustrated by simulation in Supplementary Figure S2. Furthermore, the system does not require expensive optical components such as spatial light modulators, so the cost is low. The sensitivity, accuracy and repeatability were verified with silicon wafers, engineered microspheres and fixed cells. In addition, dry cell mass remained nearly constant during osmotic pressure variations, whereas cellular size changed dramatically. This means that MWII could reveal the intrinsic cellular status without culture environment fluctuation disturbance, which is a potential merit for biological applications [30].

Nowadays, some QPI platforms lacked cell incubators [31,32] while some not [8,33]. In our system, in order to monitor long-term in situ and dynamic cell dry mass, a customized incubator was developed to control temperature, carbon dioxide concentration and humidity. Compared to commercial incubators, the rise time of temperature and carbon dioxide concentration were much less due to its far smaller volume, which helps to shorten system preparation time. Another benefit of the customized incubator was quantitative liquid or drug dosing without moving samples, which allowed for monitoring of instantaneous changes such as nuclear mechanoresponses triggered by stretch [34], as well as the response of living cells instantaneously induced by drugs [35]. Besides, infrequent sample movement facilitated long-term automatic monitoring and reduced the risk of contamination.

In our experiments, the growth characteristics between HeLa cells and HCerEpiC were compared using MWII. The dry mass of HeLa cells consistently increased before the M phase, whereas that of HCerEpiC increased and then decreased. The maximum growth rate of HeLa cells was 11.7% higher than that of HCerEpiC. These differences might be caused by the unrestricted proliferation and cycle progression of tumor cells with more surface growth factor receptors on the surface [36]. Additionally, the dry mass of the nucleus fluctuated during a cell cycle, and the nuclear dry mass peaks may be due to the entry of cell cycle regulators into the nucleus, such as cyclin B1/CDC2 [37].

The relationship between single-cell heterogeneity and chemotherapeutic efficacy was revealed for the first time with a quantitative imaging method at the ultrastructure level. We found that single cells with higher nuclear dry mass and nuclear density standard deviations were more likely to survive chemotherapy. Higher nuclear dry mass means higher nuclear protein or DNA content, which affects cell survival and chemotherapy resistance. As for surviving single cells with higher nuclear density standard deviations, a possible reason is that the genomic information spaces of those cells were expanded, and the intercellular transcriptional heterogeneities were increased, which allowed cells to stochastically develop chemotherapeutic resistance in real-time [5]. This phenomenon provides a potential novel perspective for tumor treatment.

## 5. Conclusions

We demonstrated a quantitative MWII technique for single-cell dry mass measurement during long-term culture, which could reveal intrinsic cellular conditions at an ultrastructural level. The proposed method has three main advantages over similar techniques. First, phase wrapping, which is a common problem in quantitative phase imaging techniques, was avoided with multiple illumination wavelength modifications. Thus, the phase periodicity was extended. Second, the thickness and RI information were independently reconstructed, meaning that the measurement accuracy was greatly improved for the reason that cellular dry mass measurements would not be affected by nanoscale structure fluctuations. Finally, a customized compact cell incubator was assembled into the imaging system for long-term cell culture and monitoring. Taking advantage of all above, we first performed long-term subcellular analysis using the quantitative imaging method and revealed the relationship between single-cell heterogeneity and chemotherapeutic efficacy. The results of our research revealed that MWII had significant potential applications for cell-growth dynamics research, cell-subtype analysis, drug-effect monitoring, cell-metabolism detection, cell-health characterization, medication guidance and adjuvant drug development.

## Supplementary Materials

The following are available online at https://www.mdpi.com/article/10.3390/cells10071635/s1, Figure S1: Culture performance comparison of the customized incubator (A) and the commercial incubator (B); Figure S2: (A) 3D numerical simulation diagram of ergodic method. (B) Equivalent 2D simulation result of ergodic method. The accuracy comparison between one (C) and multiple (D) wavelength measurements; Figure S3: Normalized dynamic ratio of dry mass and size for 100 living single HeLa cells at hy-poosmotic conditions; Section S1: The Customized Incubator; Section S2: The Accuracy Improvement with Multiple Wavelength Measurements; Section S3: The Sensitivity of Dry Cell Mass; Section S4: The Noise of Dry Mass Measurement; Section S5: Quantitative Dry Mass Measurement of Living Cells at Hypoosmotic conditions.
