# Supplementary material for: Label-Free and Quantitative Dry Mass Monitoring for Single Cells during In Situ Culture
Source: Cells. 2021 Jun 29;10(7):1635. doi: 10.3390/cells10071635 (PMC8303735; doi:10.3390/cells10071635)
Supplement: Supplementary file 1 [file cells-10-01635-s001.zip › cells-1225096-supplementary.pdf]

cells

MDPI

Supplementary Materials

Label-Free and Quantitative Dry Mass Monitoring for Single Cells during in Situ Culture

Ya Su †, Rongxin Fu †, Wenli Du, Han Yang, Li Ma, Xianbo Luo, Ruliang Wang, Xue Lin, Xiangyu Jin, Xiaohui Shan, Wenqi Lv and Guoliang Huang *

Department of Biomedical Engineering, School of Medicine, Tsinghua University, Beijing 100084, China; suya16@mails.tsinghua.edu.cn (Y.S.); thu_frx@mail.tsinghua.edu.cn (R.F.); dwl19@mails.tsinghua.edu.cn (W.D.); yh18@mails.tsinghua.edu.cn (H.Y.); mali20020130210@163.com (L.M.); xbluo@126.com (X.L.); wangrl13@mails.tsinghua.edu.cn (R.W.); lin-x15@mails.tsinghua.edu.cn (X.L.); jin-xy17@mails.tsinghua.edu.cn (X.J.); sxh19@mails.tsinghua.edu.cn (X.S.); lvwq20@mails.tsinghua.edu.cn (W.L.)

* Correspondence: tshgl@tsinghua.edu.cn; Tel.: +86 136 0120 6985

† These authors are contributed equally to this work and should be regarded as joint first authors.

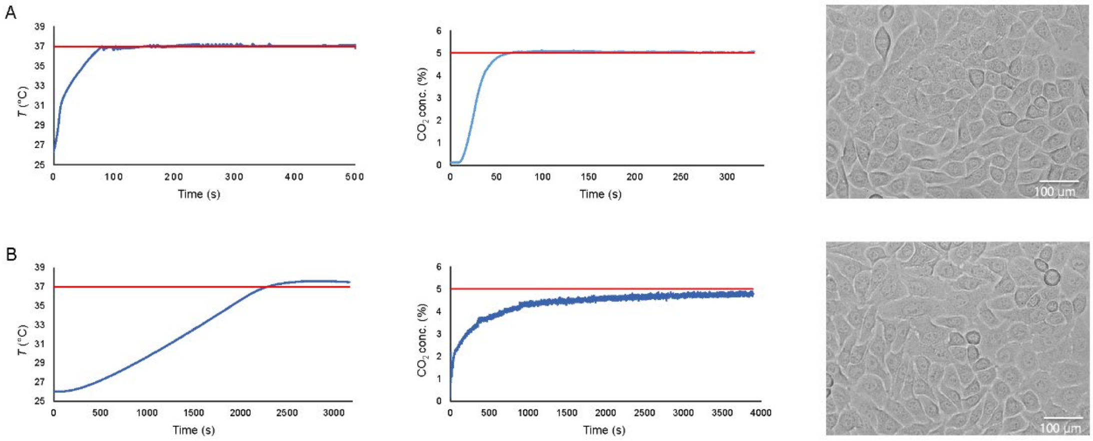

Figure S1. Culture performance comparison of the customized incubator (**A**) and the commercial incubator (**B**) (temperature curve: left; carbon dioxide concentration curve: middle; culture performance after 72 h: right). To show the control fluctuations and the steady errors, the horizontal axes of the two incubators were set to not be same.

Section S1. The Customized Incubator

The customized incubator was heated by two customized polyimide heating films buried in the upper and lower plates. Pt100 sensors were placed near the heating films, and a Wheatstone bridge was utilized to measure temperature accurately. Then, an analog–digital converter (AD7705, Analog Devices, Norwood, WA, USA) transmitted the voltage to the microcomputer (STM32f103zet6, STMicroelectronics, Geneva, Switzerland). A proportion–integration–differentiation algorithm was utilized to determine the output. The concentration of CO_2 was detected by a sensor COZIR™. When the concentration of CO_2 was below 4.9%, a pinch valve (WK04, Wokun Technology, HangZhou, China) opened for CO_2 addition. A humidity sensor (SHT75, Sensirion, Zurich, Switzerland) was utilized to monitor the incubator humidity. In the middle of the upper and lower plates, there were indium–tin oxide-coated (ITO) glasses that could be heated to prevent condensation. The incubator fit both upright and inverted microscopes.

The performance of the compact incubator was verified and compared to a commercial incubator. As shown in Figure S1, the temperature control, carbon dioxide concentration control and culture performance of the two incubators were compared. For the temperature control of the proposed incubator, the rise time was 1.2 min, and the steady error was 0.02 °C. For the temperature control of the commercial incubator, the rise time was 40

min, and the steady error was 0.50 °C. For the carbon dioxide concentration control of the proposed incubator, the rise time was 1 min, and the steady error was 0.06%. For the carbon dioxide concentration control of the commercial incubator, the rise time was 26 min, and the steady error was 0.40%. The rise time of the proposed incubator was much more rapid, and the steady errors were much smaller than that of the commercial incubator. For cell culture performance, the live rates and cellular morphologies of these two incubators were the same. This proved that the cell survivability of the compact incubator was comparable to that of a standard commercial one.

Section S2. The Accuracy Improvement with Multiple Wavelength Measurements

For common quantitative phase imaging techniques, RI measurement accuracy is usually affected by the morphology fluctuations of the sample. The intensity of the coherent item is seriously influenced by the axial position of the scattering signal. With only one illumination wavelength, this axial position could be roughly determined as the physical distance between the focus plane and the substrate, which could be recorded with an axial objective moving stage. While with four different illumination wavelengths, RI and nanoscale axial fluctuation of the scattering signal could be both calculated with the ergodic method. As in Equation (3) in the manuscript, the value of Δn and Δz could be obtained simultaneously within a reasonable range. In this work, the ergodic range of Δn was set to be −0.3 to 0.3 with the ergodic step of 0.0001. Additionally, the ergodic range of Δz was −300 nm to 300 nm with the ergodic step of 0.1 nm.

The error between actual ($I_a(\lambda)$) and theoretical ($I_t(\lambda)$) coherent intensity of the image plane is:

$$D = \sum_{\lambda} |I_a(\lambda) - I_t(\lambda)|^2, \quad \text{(S-1)}$$

D is a monotone function of Δn and Δz. In order to verify the feasibility of the algorithm, a simulation experiment was designed. As shown in Figures S2A and B, the values of Δn and Δz were obtained when D was minimum. Besides, a simulation result is shown to demonstrate the theoretical accuracy improvement. The accuracy comparison between one and multiple wavelength measurements is illustrated by simulation in Figure S2C and D. When the error of z was 1 nm, the errors of the obtained RI were 0.0079 for one wavelength measurement while 0 for multiple wavelength measurements.

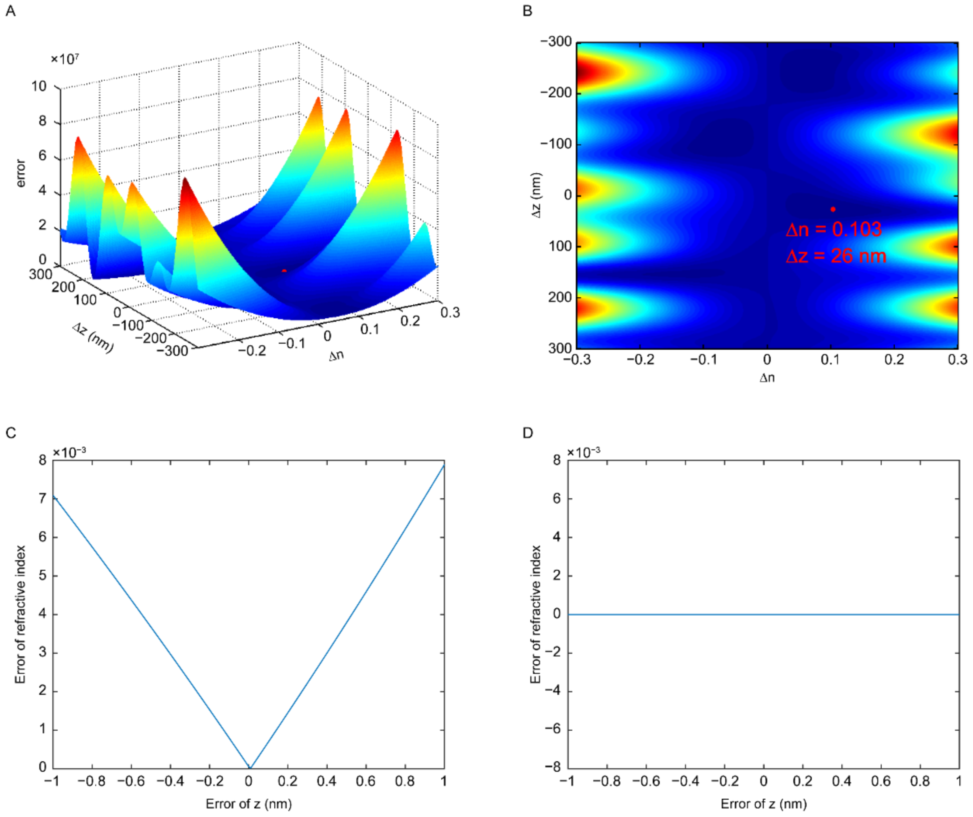

Figure S2. (**A**) 3D numerical simulation diagram of ergodic method. (**B**) Equivalent 2D simulation result of ergodic method. The accuracy comparison between one (**C**) and multiple (**D**) wavelength measurements.

Section S3. The Sensitivity of Dry Cell Mass

S_{THICK}, S_{RI}, S_{OPD} and S_{MASS} represent the thickness sensitivity, refractive index (RI) sensitivity, optical path difference (OPD) sensitivity and dry cell mass sensitivity of multichannel, wide-field interferometric imaging (MWII). In this work, the thickness sensitivity was 2.40 nm, and the OPD sensitivity of MWII equals:

$$S_{OPD} = S_{THICK} \times n_{SiO_2}, \quad \text{(S-2)}$$

where n_{SiO_2} is the average RI of silica, which is 1.45. Thus, the OPD sensitivity equals 3.48 nm. For dry cell mass measurement, the sensitivity of the cell average RI equals:

$$S_{RI} = \frac{S_{OPD}}{D}, \quad \text{(S-3)}$$

where D represents the depth of field for the microscope. According to the Gladstone–Dale relation [12], the sensitivity of cell dry mass equals:

$$S_{MASS} = \frac{S_{RI}}{\alpha} DS = \frac{S_{OPD}}{\alpha} S, \quad \text{(S-4)}$$

where α is the specific refractive increment (0.18 mL × g^{-1}) and S is the area of a pixel (0.05 μm^2). Thus, the sensitivity of cell dry mass is 3.48/0.18 × 0.05 = 0.97 fg.

Section S4. The Noise of Dry Mass Measurement

We evaluated the spatial and the temporal noise of our technique by studying the fluctuations of RI values in a region free of samples. For spatial noise, the RI standard deviation of a 512 × 512 (119 × 119 μm) pixels image was 7.13 × 10^{-4}. For temporal noise, the RI standard deviation of 200 successive acquisitions for each pixel was calculated. Additionally, the average RI standard deviation was 8.74 × 10^{-4}. The density was evaluated

using the Gladstone–Dale relation: $\rho = (n - n_w)/\alpha$, where n and n_w are the RI of sample and water, and α is the specific refractive increment (0.18 mL × g^{-1}). Therefore, the spatial and temporal noise of dry mass measurement in this work were 0.33 and 0.40 fg.

Section S5. Quantitative Dry Mass Measurement of Living Cells at Hypoosmotic conditions

The normalized dynamic ratio of dry mass and size of 100 HeLa cells were compared. The dry mass and size of HeLa cells were measured when cells were ~90% confluent. Then, 100 μL of sterile deionized water was pumped into the Petri dish within the compact cell incubator using a micropump (HALMA, Amersham, UK). Prior to that, 100 μL of culture medium was pumped out of the dish to maintain a constant culture volume of 1 mL. The cell dry mass and size were measured at this hypoosmotic condition. After perfusion and measurement were repeated five times, the cells were washed twice with PBS and then perfused with 1 mL complete medium. Cell dry mass and size were measured again at this physiological osmotic pressure. The whole experiment could be performed within 10 min, so that the changes in cell-intrinsic living status could be ignored. As shown in Figure S3, dry cell mass remained nearly constant during osmotic pressure variations, whereas cellular size changed dramatically. The average maximum relative change in cell size (27.4%) is far more than that of cell dry mass (3.3%), which proved again that MWII could monitor intrinsic cell status despite environmental changes.

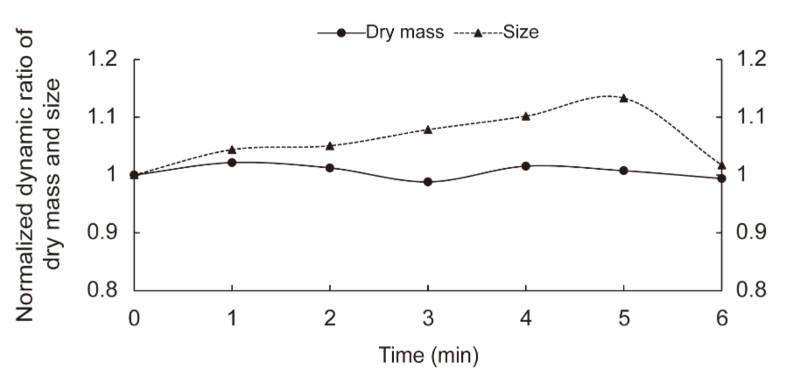

Figure S3. Normalized dynamic ratio of dry mass and size for 100 living single HeLa cells at hypoosmotic conditions.
